# Microemboli-monitoring during the acute phase of ischemic stroke: Is it worth the time?

**DOI:** 10.1186/1471-2377-10-79

**Published:** 2010-09-10

**Authors:** Titto T Idicula, Halvor Naess, Lars Thomassen

**Affiliations:** 1 Department of Neurology, Haukeland Hospital, Bergen, Norway; 2 Institute of Clinical Medicine, University of Bergen, Bergen, Norway

## Abstract

**Background:**

The prevalence of microembolic signals (MES) during the acute phase of ischemic stroke and its influence on outcome is not well studied. The aim of our study was to determine the prevalence of MES, the different factors that are associated with the presence of MES and the association between MES and outcomes in stroke patients investigated within 6 hours after the onset of ischemic stroke.

**Methods:**

We included unselected ischemic stroke patients who underwent microemboli-monitoring within six hours after stroke onset. Microemboli-monitoring of both middle cerebral arteries (MCA) was done for a period of 1 hour using 2-MHz probes applied over the trans-temporal window. Prevalence of MES, predictors for the presence of MES and the association between MES and various outcome factors were analyzed.

**Results:**

Forty patients were included. The mean age of the patients was 70 years. The prevalence of either ipsilateral or contralateral MES were 25% (n = 10). The predictors for the presence of MES were older age (OR 9; p = 0.03), higher NIHSS (OR 28; p = 0.02), intracranial stenosis (OR 10; p = 0.04) and embolic stroke (large-artery atherosclerosis and cardioembolism on TOAST classification) (OR 7; p = 0.06). MES were not independently associated with short-term functional outcome, long-term mortality or future vascular events.

**Conclusions:**

MES are moderately frequent following acute ischemic stroke. Microemboli-monitoring helps to better classify the stroke etiology. However, the presence MES did not have any prognostic significance in this study.

## Background

Previous studies have shown that microemboli to brain occurs following an ischemic stroke [1-12]. These clinically silent microemboli can be detected as microembolic signals (MES) using transcranial Doppler (TCD). There is a wide variation in the prevalence of MES after stroke. A pooled analysis of ischemic stroke patients with a known source of embolism have shown that the prevalence of MES in symptomatic ICA stenosis, asymptomatic ICA stenosis and aortic atheroma as 42%, 8% and 32% respectively [13]. However, the prevalence of MES also depends on timing of monitoring, showing higher prevalence when microemboli-monitoring is done closer to stroke onset [2,3,10]. But studies to assess the true prevalence of MES immediately following ischemic events are lacking. Also, the implications of MES in the first six hours after stroke onset is not studied before.

The common source of MES is thought to be from heart (atrial fibrillation, artificial heart valves etc.) or from an atherosclerotic plaque. While these sources are major risk factors for symptomatic thrombo-embolic events, MES per se may not cause symptoms and its clinical significance is not fully known. There is some evidence that microemboli may obstruct small arterioles and lead to subsequent white matter degeneration [14]. Therefore it is important to assess the prevalence and the significance of MES.

Previous studies have shown that intravenous anti-platelet medications can reduce microemboli [15]. Therefore it is reasonable to assume that interventions such as double platelet inhibition, anticoagulation or platelet inhibition along with anticoagulation may reduce or abolish microemboli. It will be futile to conduct such studies without knowing the true prevalence of MES in various stages of stroke, the type of stroke that are associated with MES and the significance of microemboli on stroke outcomes.

The aim of our study was to assess the prevalence of MES in acute stroke patients within 6 hours after stroke onset. We also aimed to study the etiology associated with MES and its significance on functional and survival outcomes.

## Methods

The study was conducted at Haukeland University Hospital, Bergen, Norway for a period of two years between January 2007 and December 2008. The hospital covers a well-defined geographic area with a population of 235,000. All patients were admitted to stroke unit under the department of Neurology.

In this prospective study, we included patients with ischemic stroke or TIA presented within six hours after the onset of symptoms. Patients were included whenever possible, i.e. if an experienced examiner (TTI, LT) was available, if investigation could be performed in less than six hrs after symptom onset, and if an adequate ultrasound window was found.

Microemboli-monitoring using TCD was done earliest possible according to the availability of the investigators (TTI & LT). We used Pioneer Nicolet TC 8080 for TCD examination and microemboli-monitoring. Both MCA were insonated using a trans-temporal approach. Signals were obtained from the most proximal part of MCA (usually at 56 ± 4 mm). The gain was set to the minimum possible and MES were detected automatically as "HITS" (high intensity transient signals). Sample volume length was set at 10 mm. Machine detected HITS were manually inspected to rule out artifacts. The monitoring was done continuously for one hour. Presence of one or more microemboli during the one-hour monitoring was considered MES positive. Microemboli was defined using the criteria drawn up by the International Consensus Group on Microembolus Detection[16].

All patients underwent TCD study of all segments of MCA and some patients underwent magnetic resonance angiogram of intracranial vessels. A stenosis above 50% on either test was considered positive for intracranial stenosis. TCD criteria used to define 50% stenosis was a mean velocity over 100 cm/sec.

A neurologist assessed NIHSS at the time of admission. Eligible candidates received intravenous thrombolysis according to SITS-MOST criteria [17]. Patients who were not eligible for thrombolysis were treated with antiplatelet medications after ruling out cerebral hemorrhage. The choice of antiplatelet medication was left to the treating neurologist. Risk factors were defined according to a protocol. Hypertension (HTN) was defined as treatment with antihypertensive drugs before stroke onset. Diabetes mellitus (DM) was defined as treatment with glucose lowering medications or diet prior to stroke onset, or a fasting serum glucose >7.7 mmol/L during hospital stay.

Active smoking was defined as the use of at least one cigarette per day prior to stroke onset. History of atrial fibrillation (AF), previous stroke or TIA, coronary artery disease and peripheral vascular disease was registered during hospital stay.

All patients underwent Duplex sonography of the carotid arteries. ECG was done on all patients and echocardiography was done on patients with suspected cardiac source of emboli. The etiology of stroke was classified as large-artery atherosclerosis, cardioembolism, small vessel occlusion, stroke of other determined etiology and stroke of unknown etiology based on TOAST criteria. Age was categorized as <65 and ≥ 65 years. Outcome was measured by modified Rankin Scale (mRS) 7 days after stroke onset or on discharge if discharged earlier. Poor outcome was defined as mRS ≥ 3. Survival data was obtained from the National Population Registry of Norway, where all permanent residents are registered. All causes of mortality were included. Patients were followed for a maximum of 2 years for future vascular event rate and survival analysis.

Informed consent was obtained from all the patients and the study was approved by the local ethics committee.

### Statistics

Continuous variables are expressed as mean and standard deviation for parametric variables and as median and 95%CI for non-parametric variable. Pearson's Chi-square test and Fisher exact test were used to assess odds ratio and significance for fourfold tables. Binary logistic regression was used to analyze the functional outcome. Cox-regression analysis was used for the analysis of survival and future vascular events. Statistical analysis was performed with SPSS 14.0 software.

## Results

A total of 49 patients underwent microemboli-monitoring during the two-year period. Nine (9) patients were excluded because of poor or absent ultrasound window. The remaining 40 patients were included in the study. The mean age (years) of patients with poor or absent window was higher than the mean age of patients with an adequate ultrasound window (76.4, 67.9; p = 0.12). Table 1 shows the general characteristics of the study population. Ipsilateral or contralateral intracranial stenosis was present in 6 patients (15%). The prevalence (95% CI) of MES on the ipsilateral side was 20 (7-33)%. The prevalence (95% CI) of MES on the contralateral side was 10 (11-39)%. The prevalence (95% CI) of MES on either ipsilateral or contralateral side was 25 (8-29)%. Twenty four (24) out of total 40 patients underwent thrombolysis. The prevalence of MES in those patients who underwent thrombolysis was higher than those who did not undergo thrombolysis (29.2% versus 18.8%, p = 0.7).

**Table 1:** General characteristics and the prevalence of microembolic signals of the study population (n = 40)

Prevalence of ipsilateral microemboli (95% CI)	20 (7-33)
Prevalence of contralateral micromboli (95% CI)	10 (2-20)
Prevalence of ipsilateral or contralateral microemboli (95% CI)	25 (11-39)
Mean age (SD)	70 (15.5)
Sex-male (%)	72.5
Median NIHSS (interquartile percentiles)	7 (3-12)
TIA (%)	5.1
Intracranial stenosis (%)	15
**Etiology-TOAST**	
Large-aretry atherosclerosis (%)	33.3
Cardioembolism (%)	28.2
Small vessel disease (%)	5.1
Other (%)	5.1
Unknown (%)	28.2
Thrombolysis (%)	61.5
**Pre-existing conditions**	
Stroke/TIA (%)	28.9
Atrial fibrillation (%)	16.1
Coronary artery disease (%)	15.4
Hypertension (%)	50
Diabetes mellitus (%)	10.5
Current smokers (%)	24.2
Peripheral vascular disease (%)	9.4
Migraine (%)	23.8
Depression (%)	33.3

NIHSS = National Institute of Health Stroke Scale

TOAST **= **Trial of Org 10172 in Acute Stroke Treatment

TIA = Transient ischemic attack

Table 2 shows the predictors for the prevalence of at least one MES one either ipsilateral or contralateral side in various patient subgroups. Patients older than 65 years of age were nine times more likely to have MES compared to those below 65 years (p = 0.02). Patients with severe stroke (NIHSS ≥ 14) were 28 times more likely to have MES compared to those with milder stroke severity (NIHSS < 14) (p = 0.007). Patients with intracranial stenosis were 10 times more likely to have MES compared to those without stenosis (p = 0.007). A similar analysis of isolated ipsilateral and isolated contralateral MES showed intracranial stenosis as the only significant predictor of MES (OR = 11, p = 0.006 and OR = 11, p = 0.001 respectively).

**Table 2:** Prevalence of MES in various patient subgroups along with OR for the subgroups that are significantly associated with MES (n = 40).

Group		%	OR	p-value
Age	< 65	5.9	9	0.03
	≥ 65	36.4		
Sex	Male	20.7		0.26
	Female	36.4		
Stroke type	Stroke	24.3		0.58
	TIA	0		
NIHSS	0-7	15	Ref	0.02
	8-13	8.3	14	
	> 13	71.4	28	
Intravenous thrombolysis	Yes	29.2		0.36
	No	18.8		
Etiology-TOAST	Embolic	33.3	7	0.06
	Non-embolic	12.5		
Intracranial stenosis	No	16.7	10	0.04
	Yes	66.7		
Previous stroke/TIA	No	14.8	4.8	0.06
	Yes	45.5		
Atrial fibrillation	No	23.1		0.13
	yes	60		
Active Smokers	No	28		0.6
	yes	25		

* OR calculated using Chi-square for dichotomous variables and logistic regression for polytomous variables.

NIHSS = National Institute of Health Stroke Scale

TOAST **= **Trial of Org 10172 in Acute Stroke Treatment

TIA = Transient ischemic attack

**Figure 1:**
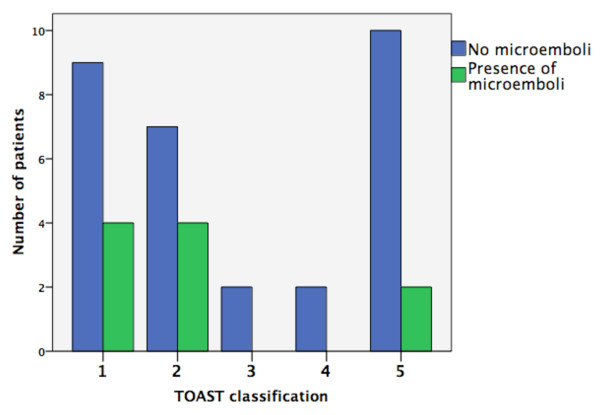
**Presence of microemboli and stroke etiology according to TOAST classification**. 1 = Large-artery atherosclerosis. 2 = Cardioembolism. 3 = Lacunar. 4 = Other. 5 = Unknown

Patients with embolic etiology (cardioembolism or large-artery atherosclerosis) were seven times more likely to have MES compared to other etiologies (small vessel, unknown cause and other causes) based on TOAST criteria (p = 0.03). Eight out of ten patients with MES had an embolic source for stroke. The prevalence of MES in patients with embolic etiology was 33.3% and that of non-embolic etiology was 12.5%. None of the patients with lacunar stroke or stroke from 'other etiology' (one patient with moyamoya disease and one patient with carotid dissection) had MES. Out of nineteen patients with `unknown etiology`, two patients with insufficient stroke work-up had MES. Figure 1 shows the prevalence of MES in various TOAST subgroups. Sex, stroke type (TIA vs. stroke), smoking habit and pre-existing atrial fibrillation were not associated with the presence of MES.

On univariate logistic regression analysis, MES were significantly associated with poor functional outcome (OR 9, p = 0.017). When the analysis was done after adjusting for the confounding factors (age, sex and NIHSS), the association was no more significant (OR 3.4, p = 0.25). Isolated ipsilateral MES and isolated contralateral MES were also not associated with functional outcomes (OR = 4.3, p = 0.26; OR = 0.95, p = 0.13)

**Figure 2:**
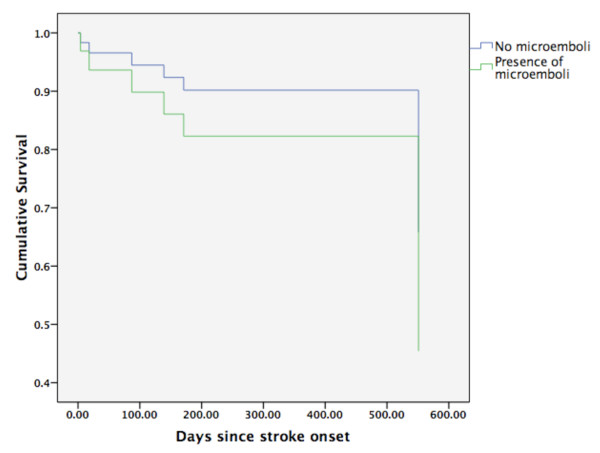
**Survival pattern of patients with and without MES at admission (n = 40)**. *Cox-regression survival curve after adjusting for age, sex and NIHSS.

Six patients died during the study period of two years. On Cox regression analysis, after adjusting for age, sex and NIHSS, presence of MES were not associated with mortality (OR 1.9, p = 0.55). Figure 2 shows the survival curve for patients with and without MES. Two patients had recurrent ischemic events. None of these patients had MES at index stroke.

## Discussion

Our study shows that the prevalence of MES in stroke patients with thrombo-embolic etiology is moderately frequent while the prevalence of MES in non-thromboembolic stroke is infrequent in the first six hours after stroke. One third of the stroke patients with embolic etiology had MES while only one eighth of the stroke patients with non-embolic etiology had MES. Eight out of ten patients with MES had either large-artery or cardio-embolic etiology.

Detection of the source of emboli is important in preventing future strokes. Based on the present stroke classifications such as the TOAST criteria, more than one third of the patients falls under the subgroup of 'undetermined etiology' [18,19]. In two previous studies, the prevalence of MES in stroke patients with embolic etiology (large artery and cardioembolic) was 10% and 43% while those of non-embolic etiology were 2.9% 19% [7,8]. The prevalence of MES in lacunar stroke in those studies were 0% and 17.6%. In our study the prevalence of MES in embolic, non-embolic (including lacunar) and lacunar stroke were 33.3%, 12.5% and 0% respectively. A similar prevalence was also observed in the sub-group of patients who underwent thrombolysis (37.5% in embolic etiology and 12.5% in non-embolic etiology). Thus, detection of MES helps us to better classify the etiology of stroke. We may assume that MES is moderately sensitive, but highly specific, in detecting embolic stroke based on the findings from this study as well as the previous studies [7,8].

The prevalence of microemboli among patients who underwent thrombolysis was higher than in those who did not undergo thrombolysis (29.2% versus18.8%), however did not reach statistical significance (p = 0.36). It is argued that MES in patients who undergo thrombolysis may represent fragmentation of thrombus proximal to insonation by thrombolytic agents. A clear conclusion cannot be drawn from our study because the association was not statistically significant.

One of the issues in performing microemboli-monitoring immediately following stroke onset is that it requires considerable time and manpower to set up the machine and to perform monitoring constantly. Unless there is a clear and superior benefit in detecting more patients with MES or in predicting outcomes, it is futile to perform the time-consuming microemboli-monitoring during the phase immediately following stroke. A prevalence of 25% as shown in our study is not considerably higher than in other studies in which monitoring was done beyond the first six hours after stroke onset. Our study also failed to shows any association between MES detected during the acute phase of stroke and stroke outcomes (functional outcome and survival) or future vascular events. Thus, the difficulty of performing microemboli-monitoring along with its uncertain association with stroke outcomes and future vascular events questions its clinical utility during the acute phase of stroke. However, this interpretation needs to be taken cautiously since ours is a single-center study of non-consecutive stroke patients. This study may be underpowered to assess the outcomes. Therefore the results need to be confirmed with larger studies. Also, the absence of prognostic significance may not apply to certain specific sub-groups such as symptomatic carotid stenosis, especially those with unstable carotid plaque [20].

This study has some limitations. We did not include all consecutive patients admitted within the first six hours after the stroke. Therefore, the prevalence rate in our study may not represent the actual prevalence. Microemboli-monitoring was done only once. Multiple monitoring during successive days following stroke might yield more patients with microemboli. A single monitoring also makes it difficult to assess the source of microemboli in those patients with intracranial stenosis because microemboli could have originated from a resolving thrombus. Another limitation is a relatively small patient population. A large study, however, may be practically difficult to conduct during the immediate phase following stroke onset.

## Conclusions

Microemboli-monitoring following acute ischemic stroke helps to better classify the etiology of stroke as embolic versus non-embolic. The usefulness of microemboli-monitoring immediately following stroke onset for prognostication seems questionable.

## Competing interests

The authors declare that they have no competing interests.

## Authors' contributions

**TTI & LT**: Participated in the design of the study, participated in analysis and interpretation of data, participated in manuscript drafting. **HN**: Participated in the design of the study, participated in data collection.

All the authors have read and approved the final manuscript.

## Pre-publication history

The pre-publication history for this paper can be accessed here:

http://www.biomedcentral.com/1471-2377/10/79/prepub
